# Implementation and evaluation of Illness Management and Recovery (IMR) in mandated forensic psychiatric care – Study protocol for a multicenter cluster randomized trial

**DOI:** 10.1016/j.conctc.2022.100907

**Published:** 2022-03-09

**Authors:** Peter Andersson, Malin Tistad, Åsa Eriksson, Pia Enebrink, Knut Sturidsson

**Affiliations:** a Division of Psychology, Department of Clinical Neuroscience, Karolinska Institutet, 17177, Stockholm, Sweden; b Centre for Clinical Research Dalarna, Uppsala University, Nissers väg 3, 791 82, Falun, Sweden; c School of Health and Welfare, Dalarna University, 791 88, Falun, Sweden; d Division of Family Medicine and Primary Care, Department of Neurobiology, Care Sciences and Society, Karolinska Institutet, 14183, Huddinge, Sweden

**Keywords:** Forensic mental health, Illness management and recovery, Schizophrenia spectrum disorder, Offender rehabilitation

## Abstract

**Introduction:**

Forensic mental health care is hampered by lack of evidence-based treatments. The Swedish forensic mental health population consists of patients suffering from severe illnesses such as schizophrenia and bipolar disorders, similar to populations in international studies. Illness Management and Recovery (IMR) is an intervention for patients with serious mental illness, based on psychoeducational, cognitive-behavioral and motivational components. The purpose is to strengthen participants’ illness management skills and recovery.

**Objective:**

To test effectiveness of IMR within forensic mental health by comparing it to treatment as usual.

**Method:**

This is a cluster-randomized controlled trial. Patients in forensic mental health inpatient units are randomized to an active (IMR) or a control condition (treatment as usual). Clustering of patients is based on ward-units where inpatients are admitted. Patients in the active condition receive two group and one individual IMR sessions per week. The treatment phase is estimated to last nine months. Outcomes include illness related disability, illness management skills, sense of recovery, hope, mental health and security related problems. Outcomes are measured at baseline, four months into treatment, at treatment completion and at three months follow-up. Staff experiences of implementing IMR will be explored by a self-report measure and semi-structured interview based on Normalization Process Theory.

**Ethics and dissemination:**

The study is approved by the Swedish Ethical Review Authority (Registration No. 2020–02046). Participation will be voluntary based on written informed consent. Results will be disseminated through peer-reviewed articles and conferences. The study is registered in the US registry of clinical trials (NCT04695132).

## 1 Introduction

Previous research has highlighted difficulties associated with treating criminal offenders suffering from serious mental illness. One fundamental aspect of forensic mental health care is that successful care does not only mean alleviating effects of mental illness, but also include minimizing risk of criminal recidivism. In addition to this, forensic mental health patients often suffer from psychiatric comorbidity and behavioral or lifestyle-associated problems that complicate treatment [[1], [2], [3]]. Furthermore, the compulsory nature of care complicates caregiver-caretaker relationships [4,5]. Overall, there is currently a paucity of knowledge guiding efforts to address needs of forensic mental health patients [1,6]. Considering the suffering associated with severe mental illnesses and the ramifications criminal re-offense have for society, this is an area in need of attention.

In a Swedish context, official data indicate that a majority of patients in forensic mental health services suffer from a schizophrenia spectrum disorder or a bipolar syndrome. Psychiatric comorbidity is common [46]. Furthermore, several governmental reviews conclude that scientific support for non-pharmacological interventions is highly unsatisfactory. These reviews also point to a large degree of heterogeneity in care provided at different in-patient forensic mental health facilities. One common practice is to import interventions from non-forensic psychiatry. However, translatability of interventions from a non-forensic context cannot be taken for granted and studying treatment effects specifically in forensic mental health populations is recommended [8,44]. With this in mind, we aim to study the effectiveness of a psychosocial treatment program, Illness Management and Recovery (IMR), within forensic mental health in-patient care. IMR was designed for individuals suffering from severe mental health problems [[9], [41]]. Previous research has focused on non-forensic populations, suffering mainly from schizophrenia spectrum disorders but also including patients with other syndromes (e.g. [[11], [12], [40]]). In Sweden, psychosocial treatments such as IMR, are strongly recommended in clinical guidelines for treatment of patients with schizophrenia spectrum disorders [45]. Given the aforementioned clinical characteristics of the forensic population, it is hardly surprising that IMR has started to proliferate as a treatment method within Swedish forensic inpatient clinics. Two of the authors of this study (PA & KS) have conducted a smaller pilot study of IMR (data not yet published) at a Swedish forensic clinic. However, that study focused on feasibility and acceptability, and to our knowledge, no study focusing on effects of IMR on forensic inpatients in Sweden or elsewhere has yet been published. With this in mind, we plan to evaluate the effectiveness of IMR by conducting a multi-center cluster randomized study in forensic inpatient psychiatry.

The two main objectives are:

1. To evaluate whether forensic patients receiving IMR show improvements on outcomes such as functional impairment, illness management ability, subjective recovery, sense of hope, and/or self-experienced functional status, when compared to a control group receiving treatment as usual.
2. To evaluate to what extent the presence of background risk markers for criminal recidivism and/or the functional status of participants in the treatment group moderate effects of IMR on treatment outcome measures.

In addition, a secondary objective is:

3. To explore how staff working with IMR experience the process of implementing the treatment.

## 2 Methods

**Figure 1:**
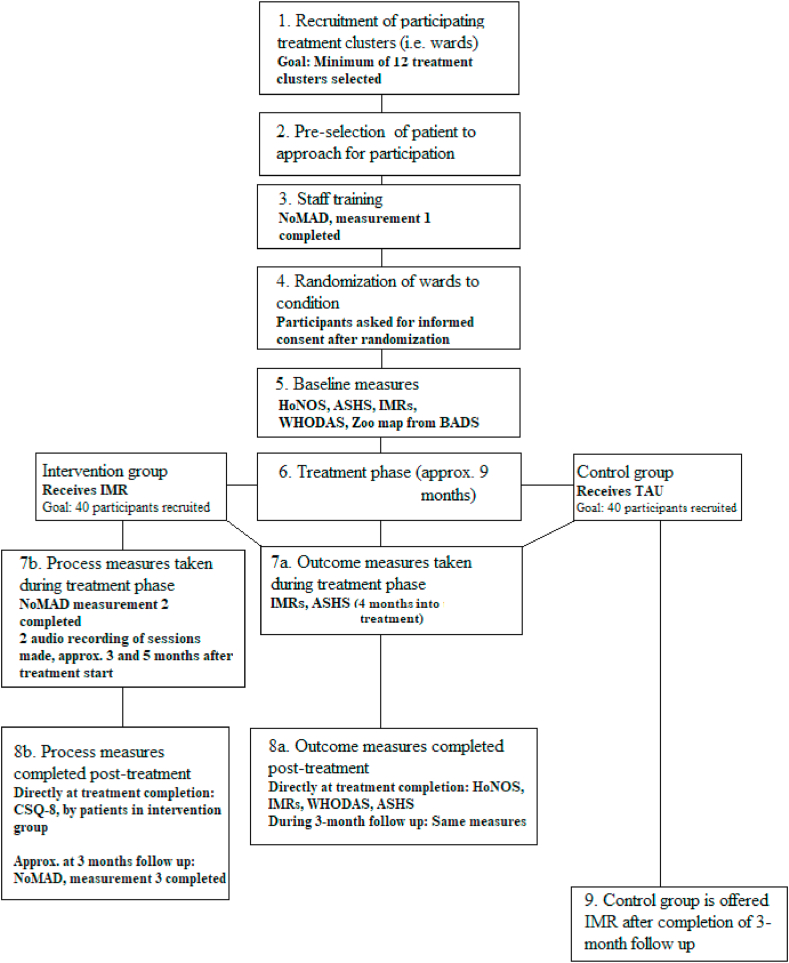
Overview of study plan and timetable.

The overall study design (described in further detail below and outlined in Fig. 1) is a cluster randomized trial conducted within inpatient forensic psychiatric clinics in Sweden. A clinic participating in the trial will nominate an even number of wards, which is the clusters of participating patients (conditional on them giving informed consent) that will be randomized to either a control or an active condition. Participants in the control condition will receive treatment as usual, whilst participants in the active condition receive IMR. Outcome measurements will be taken at four time points, baseline before treatment, four months into treatment, at treatment completion and at three months after treatment completion.

In addition to this, a mixed methods approach will be used to explore the experiences of staff delivering the IMR-treatment. This part of the study will be based on the framework postulated in Normalization Process Theory [13]. At three time-points, after completion of training, three months and twelve months after treatment commencement, staff will answer a questionnaire pertaining to implementation processes and, during the latter two assessment points, participate in semi-structured interviews regarding their experiences working with IMR.

### 2.1 Settings of the study

The planned study will be conducted within in-patient forensic mental health facilities in Sweden. These facilities are tasked with providing court mandated compulsory forensic mental health care for individuals convicted of crimes who have been found to suffer from serious mental illness during the perpetration of their offense and at the time of a subsequent forensic mental health investigation. On the basis of these criteria, a court can convict an individual to compulsory mental health care rather than a traditional prison sentence [43]. In general, a forensic mental health inpatient facility in Sweden is structured around different ward units where patients live and receive care. The typical treatment process usually starts on an inpatient basis but can later on be converted to outpatient treatment. Treatment typically spans several years and thus, most commonly an individual sentenced to forensic mental health care within the Swedish system will spend a substantial time with the in-patient ward as their main living environment [14]. Previous studies have shown that the daily life of patients at these wards are to a large extent characterized by unstructured and unplanned activities, rather than treatment per se [15].

### 2.2 Design

The project will be conducted with a cluster randomized study design. Each participating forensic mental health in-patient facility will nominate an even number of wards (i.e. clusters of patients), which will then be randomized to either the active (IMR) or control condition (treatment as usual). Randomization of clusters to condition will be matched, in the sense that each participating in-patient facility will have the same number of clusters within the two conditions (e.g. if four wards take part in the study at one facility, two wards will be randomized to each condition). Recruitment of new participating clusters of patients will take place continually until the desired sample size is reached. Assessments of patient outcome measures take place during four different time-points including baseline measurements.

In addition to the patient focused research, the study also aims to explore how staff implementing IMR experience working with the treatment method. This will be done in a mixed-methods fashion, based on the theoretical framework stipulated in Normalization Process Theory [[13], [42]]. Staff working at wards where IMR is implemented will be asked to complete a questionnaire pertaining to aspects of working with the intervention and queried about the same topic in more in-depth qualitative interviews. Data collection from staff will take place at three time-points during the study.

More details on the specifics of data collection is described below, under the heading “Measurement methods”.

#### 2.2.1 Participants and recruitment

We aim to include patients from several forensic mental health inpatient facilities in Sweden. Thus, recruitment will start with approaching the management at the forensic mental health inpatient facility, informing them about the contents of the study and inquiring about their interest in participating. If the decision is made on the clinic level to participate in the study, the representatives of the inpatient facility will be asked to nominate an even number of wards that are considered as comparable to each other as possible. These wards are the clusters of patients that will be randomized to either of the two study conditions. An equal number of wards per inpatient facility will be randomized to either one of the two conditions. In practice, this means that a necessary criterion for each in-patient facility participating in the study is that they have at least two different wards that are considered equal enough in terms of structure and patient characteristics to be comparable to each other. We aim to recruit twelve wards (clusters) for the study (six in each condition) and estimate that a total of 80 patients, 40 in each condition, consent to study participation. To achieve this, we aim to include 4–6 forensic inpatient clinics in the study.

Prior to randomization of a ward to a specific condition, the patients to subsequently be approached for participation in the study will be identified in cooperation with the clinical team according to the inclusion and exclusion criteria delineated below. After randomization patients fulfilling inclusion criteria at these wards will then be informed about the study and asked for written consent regarding their participation. Recruitment of patients at each ward unit will take place during a time limited window of approximately 3–4 weeks after randomization of the ward unit, before IMR treatment start at wards randomized to the active condition. Given the aforementioned lack of evidence-based interventions in forensic mental health care, we would consider even small effect sizes to be of relevance. With that in mind, we would have preferred a larger sample size to have a sufficiently high probability of discovering such effects. A power calculation conducted in R version 4.1.2. indicated that a total of 228 patients (19 in each cluster) would be needed to achieve 80% power to detect a true effect size of .4, if a small intercluster correlation was presumed. However, it should be noted that some studies of the treatment outcome of IMR have reported substantially higher effect sizes than 0.4 [[16], [40]].

However, the selection of our planned sample size has been made based on a trade-off between a desire for statistical power and pragmatic considerations of what is feasible in a situation of limited time and resources. Furthermore, our study can hopefully be a part of data underlying future meta-analytic studies of the efficacy of treatments within the forensic inpatient context.

In addition to patients participating in the study, staff administering IMR-treatment to patients will also be recruited as participants to the study. These staff participants will answer questions pertaining to their experiences of implementing the intervention. Staff participants will only be recruited from the wards where IMR is implemented. Recruitment of staff participants will take place after the end of staff training prior to treatment commencement. The specific composition of this sample is dependent upon the organization of the wards participating in the study, but it is expected that this will mainly consist of nurses and auxiliary staff. More in-depth inclusion and exclusion criteria both for patient and staff participants are defined below.

#### 2.2.2 Patient inclusion criteria

1. The patient is sentenced to in-patient forensic mental health care, in accordance with the Swedish judicial framework. In practice, this means that they have committed a crime warranting incarceration and that they have been found to suffer from serious mental illness in a forensic psychiatric evaluation.
2. The treating psychiatrist and treatment team at each ward must approve of participation before the potentially participating patient is given information on the study. They will be instructed to base this approval on the basis of whether or not this patient can participate in IMR and is capable of giving informed consent. Examples of such factors rendering informed consent and thus participation not possible include presence of severe active psychotic symptomatology or severe intellectual impairment. The treating psychiatrist and treatment team will have ultimate veto rights if they for some other reason think that the participation of a patient in the project is not advisable on ethical grounds or because of mental health considerations.

We do not require that study participants fulfill any specific diagnostic criteria. Given that a large majority of patients within the Swedish forensic mental health services suffers from either a schizophrenia spectrum or bipolar disorder we consider it highly likely that a majority of our sample will present these disorders as their main diagnosis. However, it is also likely that some of the patients in our sample will have autism or mild intellectual impairment as their main diagnosis. This is in line with previous research on IMR, where a majority of patients included has suffered from either schizophrenia spectrum or bipolar disorders, and a minority of participants has suffered from other forms of mental illness (see for example [11,12,16]).

#### 2.2.3 Staff inclusion criteria

1. The only requirement for staff participating in the part of the study focused on implementation processes is that staff participants are directly involved in administering IMR treatment and that they give informed consent to study participation. Participation in the study will not be a requirement for staff administering the treatment.

#### 2.2.4 Intervention administered to patients in active condition (IMR)

IMR was developed as a treatment method targeted towards people with severe mental illness, within the framework of the Evidence-Based Practice Implementation Project [9]. It is a curriculum-based program combining elements from evidence-based practices such as cognitive-behavioral therapy, motivational interviewing and psychoeducational practices, in an effort to help patients achieve better illness self-management and subjectively meaningful recovery goals. The curriculum is organized around chapters pertaining to subjects such as social support, problem solving, medication, subjective recovery and relapse prevention. These chapters involve both educational material discussed during treatment sessions and material suitable as homework assigned for in-between session work [9]. Some adaptions have been made to the material to ensure the suitability of the treatment to the forensic setting. These include exclusion of two chapters in the original treatment program. These two chapters deal with healthy lifestyle choices and navigating the health care system. The first of these chapters has yet not been translated to Swedish in an adequate fashion and the second was deemed not relevant to the Swedish context. Instead, a special chapter has been written explaining the process of forensic mental health care from a patient perspective, adapted to the applicable regulatory framework. In the other chapters included in the study, certain examples and practical exercises have been modified to ensure applicability within the forensic context. Adaptions of the original material were made as part of a pilot study (not yet published) within a Swedish forensic mental health in-patient facility. Prior to this pilot study, permission for these adaptions was obtained by the developers of IMR.

Treatment is planned to take place during two group meetings and one individual follow-up meeting per week. Group meetings will be used to discuss and work with new treatment material, share experiences amongst group members, monitor progress towards subjectively meaningful recovery goals and set homework assignments for the next group session. Individual sessions will be used to repeat and clarify information given at group sessions, tailor homework to the individual patient, support completion of homework and efforts towards achieving recovery goals. The IMR material does not state a set number of sessions for the treatment, instead the material is worked through in a pace adapted to the patients receiving treatment. However, based on our experience we estimate that the active treatment phase will last for approximately nine months. Treatment sessions will be led by the forensic mental health staff at the wards participating in the active condition (i.e. psychiatric nurses and auxiliary psychiatric care-givers). Staff administering IMR-treatment will receive two full days of training in the method, as well as a weekly supervision session during the treatment period. Training in IMR will consist of lecture style presentation of information about treatment goals and contents as well as practical exercises in using methods included in the treatment material. During training sessions, participating personnel will also be encouraged to discuss how IMR can be optimally integrated in the daily routine of clinical work. The supervision sessions will consist of 1 h weekly meetings in a group setting, focusing on solving problems emerging in treatment as well as rehearsing and emphasizing treatment goals. This training and supervision will be provided by members of the research team, mainly KS and PA. During the treatment phase, responsibility for practically leading and implementing IMR will be the staff leading and planning daily work at the ward in question (i.e. the person or persons holding leadership positions at the ward participating in the treatment condition). The caseload of individual treatment providers could vary somewhat depending on variability in staffing and local decisions regarding the division of tasks at individual treatment sites. Based on the length of the IMR-program and estimations of staffing at an inpatient facility, we estimate that the mean number of group and individual session provided by individual staff at wards in the active condition will be approximately 5 and 10, respectively. The IMR treatment integrity scale (IT-IS) [17] will be used to assess treatment fidelity in the active condition, which is described in further detail below.

#### 2.2.5 Control condition

Participants in the control condition will be given treatment as usual (TAU) during the study period. After completion of the study, participants in the control conditions will be offered participation in IMR if this is found more effective than TAU, conditional on them still being in-patients at the facilities participating in the study. Since the study will be conducted at different treatment sites, standard treatment given to participants in the control condition might differ somewhat. The TAU at each control site will be documented. In some cases, staff working at wards in the active condition and who thus have received training in IMR, might sometimes work at a ward in the control condition (for instance, in situations when there is a need to solve a shortage of staff).

#### 2.2.6 Measures to avoid contamination

To avoid contamination between the active and control conditions, we will avoid training staff that regularly work at both active and control clusters. Some instances of staff trained in IMR working at control wards can probably not be avoided (e.g. in instances were acute staff shortages have to be addressed). Since IMR is a structured group intervention, this will not mean that participants receive the intervention in its intended group format. However, one clear risk is that staff trained in IMR apply the interventions and material from IMR in an ad hoc manner. To avoid this, trained staff will be instructed not to use IMR materials or interventions in cases where they are working at control wards. Instances of staff trained in IMR working at control wards will be documented. Staff at active and control wards will be instructed not to share information on the intervention during the trial and will be given a clear rationale for this. Furthermore, management at control wards will be interviewed about the contents of care at the ward during the trial period after the conclusion of the study, to assess whether or not substantial contamination occurred.

### 2.3 Measurements

Assessment of outcome measures will be conducted at four time-points (at baseline, four-months into treatment, upon treatment completion and three months after treatment completion, see Table 1). To gather data pertaining to our secondary objective, semi-structured interviews as well as self-report measures completed by staff working at wards in the active condition will be collected. Assessment of the experiences of staff administering IMR will be gathered after completion of training but before treatment commencement, three months into treatment and a year after treatment commencement. In addition to this, audio from two IMR-sessions per group in the active cluster will be recorded and assessed with a fidelity measure. More details on the measures used in the study is outlined below.

**Table 1:** Overview of evaluation plan.

		**Timepoint**			
		**Baseline (pre-treatment)**	**During treatment**	**Treatment completion**	**Post-treatment follow up**
**Outcome measures**	WHODAS	X		X*	X
	IMRs	X	X	X	X
	ASHS	X	X	X	X
	HoNOS-S	X		X*	X
**Process measures**	IT-IS		X		
	NoMAD	X	X		X

**IMRs** = Illness management and recovery scale, **ASHS** = Adult State Hope Scale, **WHODAS**= World Health Organization Disability Assessment Schedule, **HoNOS-S**= Health of Nation Outcome Scale-Secure, **IT-IS**= IMR Treatment Integrity Scale, **NoMAD**=Normalization Process Theory Measure. **IMRs** and **WHODAS** include both client and clinician rated versions of these scales. Post-treatment follow up is planned at three months after treatment completion. Time points for measurement during treatment differs between outcome measures and process measures. * = Denotes a primary outcome measure.

#### 2.3.1 Background factors of the participants assessed before treatment commencement

To describe our sample and explore potential moderators of treatment outcome, information on some background characteristics of our sample will be collected, as described below.

#### 2.3.2 Demographic measures

Participant's age, sex, diagnoses and when the participant underwent a forensic psychiatric evaluation and was admitted to the forensic mental health services will be assessed through extracting this data from the participants’ medical records.

#### 2.3.3 Static criminogenic risk

With the aim of partially answering the second of our main objectives, the presence of static (i.e. historical) risk factors for criminal recidivism will be assessed through the medical records of participating patients. The static criminogenic risk factors assessed include the patient's number of previous convictions before admission into the forensic mental health services, index crime (most severe offense) in the trial that resulted in admission, age at first known criminal conviction, known history of substance abuse and whether or not there is evidence of criminality before the first known onset of symptoms of serious mental illness. Serious mental illness in this context refers to the main diagnosis that was assigned to the patient at the forensic psychiatric evaluation (in most cases this will mean before onset of first known active psychosis). Earlier research has indicated that such aspects can indicate increased risk of criminal recidivism and/or presence of traditional antisocial behavior patterns amongst offenders with serious mental illness [18,19].

#### 2.3.4 Clinician assessed functional status

To further address the second of our main objectives, the functional status of participants in the active group will also be assessed, with anamnestic measures of functional status as well as a shorter neuropsychological test. The background measures pertaining to functional status are focused on historical role achievement/fulfillment. These include information on how the participants have earned an income before admission into forensic services, what their living condition was, their frequency of social contacts, educational attainment and relationship status. During the first assessment of the outcome measures (before treatment commencement) the participants will also complete a short neuropsychological test called the Zoo map, which is a subtest from the Behavioral Assessment of Dysexecutive Syndrome (BADS). The Zoo map subtest is designed to measure the ability to plan and monitor one's performance and compliance with pre-specified rules [20]. The person taking the test is tasked with drawing a route throughout a zoo, whilst complying with certain rules of how the route can be designed. Earlier research has shown that persons suffering from schizophrenia show impairments in this test, when compared to healthy controls [21]. Differences between persons suffering from schizophrenia with either acute symptomatology or in a more stable phase of disease progression have also been demonstrated [22]. In addition to this, participants will also be asked to rate their own performance, by answering four questions on a four-point scale. We expect that the administration of the Zoo test will add 5–10 min to the first assessment of outcome measures.

#### 2.3.5 Outcome measures

Participant's self-rating.

1. Level of illness-related disability

Functional impairment is measured by the 36-item interview version of the World Health Organization Disability Assessment Schedule 2.0 (WHODAS 2.0). This measure assesses respondent functioning in six domains: Cognition (six items), Mobility (five items), Self-care (four items), Getting along with people (five items), Life activities (four items), Household (four items) and Participation (eight items). WHODAS 2.0 has demonstrated reliability, validity and sensitivity to change in samples with psychotic illnesses as well as a wide variety of both psychiatric and non-psychiatric illnesses [23,24]. In the Swedish context, a previous study with post-stroke patients has shown high levels of overall internal consistency and moderate to high levels of internal consistency on five of the six subscales, with “Getting along with people” being the one subscale showing non-satisfactory consistency [25]. The interview administered WHODAS 2.0 at treatment completion is one of the studies two primary outcome measures.

2. Illness management skill and sense of recovery

To assess participants' illness management skills and sense of subjective recovery the self-respondent version of the Illness Management and Recovery Scale (IMRs) will be used. This is an instrument specifically designed to assess successful treatment outcome in patients participating in IMR. It consists of 15 items, scored on five-point scale. The items generate a total score and scores on three subscales, illness management ability, sense of recovery and substance use [26]. The Swedish version of the scale has demonstrated satisfactory internal reliability, strong test-retest reliability and convergent validity with other measures of symptoms, sense of recovery and quality of life, in a sample of Swedish respondents suffering from schizophrenia or schizoaffective disorder [10].

3. Hope

To assess participants' levels of hope the Adult State Hope Scale (ASHS) will be used. This is a self-rating scale constructed to measure a respondent's level of hope by six items, answered on an eight-point scale [27]. This measure has been used in previous studies of IMR, and positive effects of the treatment have been demonstrated [28]. A previous study also demonstrated a correlation between the activity levels of schizophrenic patients participating in IMR and the ASHS [29].  

Clinician's rating.

1. Mental health and security related problems

To assess the participant's mental health and the extent of security related problems affecting them, the Health of the Nation Outcome Scale-Secure version (HoNOS-S) [30,31] will be used. This is a questionnaire consisting of 19 items rated by a clinician on a five-point scale. Twelve clinical items assess the extent of the patient problems in four areas: behavior, function, symptoms and social adaption. In addition to the twelve clinical items in the original version, the secure version of HoNOS [31] also contain seven items that focus on the need for special security measures in conjunction with the care provided for the patient rated. In this project, the participants treating psychiatrists will be asked to answer this questionnaire. The HoNOS-S administered at treatment completion is the second primary outcome measure in the study.

2. Level of illness-related disability

In addition to the interview version of WHODAS 2.0, the 36-item proxy-administered version of WHODAS 2.0 will be used. In this version an informant with good knowledge of the person assessed is asked to answer the same questions posed in the interview version of WHODAS 2.0, with necessary revisions to wording made to adapt it to the third person perspective of the proxy respondent [23]. This scale will be answered by a member of the forensic mental health staff with good knowledge of the participant.

3. Illness management skill and sense of recovery

Similar to WHODAS, IMRs also exist in a proxy-answered version. This version of the scale consists of the same number of items (15) answered on the same five-point scale as the self-report version of the questionnaire [26]. The same proxy-respondent answering WHODAS will be asked to answer the proxy-version of IMRs.

#### 2.3.6 Process related measures

1. Implementation and normalization of the treatment method

To assess the implementation of IMR and the experiences of staff working with IMR, the S-NoMAD [32] will be used. This will be completed during three time-points, before the start of the intervention (in conjunction with staff-training), three months after treatment commencement and twelve months after treatment commencement. S-NoMAD is a questionnaire designed around the theoretical framework and constructs stipulated within Normalization Process Theory (NPT). NPT is a mid-level sociological theory concerned with explaining implementation processes within the health-care sector [13]. S-NoMAD is a Swedish version of the original British version of NoMAD [33,34], that has been translated to Swedish and validated in samples of Swedish health care workers [32].

Within NPT, implementation processes are conceptualized as dependent on four different generative mechanisms, Coherence, Cognitive Participation, Collective Action and Reflexive Monitoring [13].

S-NoMAD is intended to measure the extent to which health-care personnel engage in actions of these four types. Thus, the questionnaire generates scores on four indexes of these actions. The S-NoMAD questionnaire is divided into three section, A, B and C. In addition to a few items collecting background information about the respondents’ roles within the organizational context (Section A), the questionnaire contains 3 general (Section B) and 20 specific (Section C) questions on the intervention in question. Items on Section B are answered on a 10-point scale, whilst items on Section C are answered on a 5-point scale [32]. As stipulated by the creators of the instrument, we have made certain adaptions to item wording to fit the way we use the S-NoMAD. Mainly these consist of adding the name of the intervention studied, but also include temporal aspects based upon the time point when an assessment is made (i.e. if an assessment is made before or after the IMR intervention has been initiated). With the purpose of generating more in-depth qualitative data on the implementation process, we will also conduct two follow-up interviews with staff in conjunction with the two latter assessment points when S-NoMAD is answered. These interviews will be based on the questions asked in S-NoMAD, with the purpose of giving an opportunity for interviewees to more freely expand upon their reasoning. The interviews will be conducted in a semi-structured manner. A member of the research team will conduct these interviews.

2. Quality of treatment delivered

As previously mentioned, the aim is to achieve treatment fidelity by training staff and providing access to weekly supervision. To assess how well treatment fidelity is achieved, audio recordings of IMR session will be made. We plan to record two sessions each from the wards participating in the active treatment conditions. These audio recordings will then be scored for treatment fidelity by the research team. Scoring will be based on the instrument Illness Management and Recovery Treatment Integrity Scale (IT-IS) [17]. This is a rating scale specifically designed to assess the quality of the delivery of the IMR-intervention. The scale is focused on the fidelity to the treatment manual and the strategies specified within. IT-IS is designed to be used by an IMR-proficient supervisor or expert, who, observing a session or material from a session, assesses treatment quality by rating the observed material on 16 items (13 mandatory, 3 optional items) graded on a five point scale. The audio recordings are planned to take place approximately three and five months into treatment.

3. Participants’ satisfaction with IMR

To assess the satisfaction with IMR amongst participants in the active group, they will be asked to complete the Client Satisfaction Questionnaire-8 [35] during outcome assessment post-treatment. This is a questionnaire intended to measure clients’ satisfaction with eight items scored on a scale ranging from 1 to 4. Previous studies using the instrument have demonstrated high internal reliability, good construct validity and that the items on the scale load on a single satisfaction factor [36,37].

### 2.4 Data analysis plan

Comparisons of primary outcome measures between the two conditions will be made on an intention to treat (ITT) basis. Linear mixed modeling (LMM) will be used to assess main effects of time, treatment and time by treatment interactions. A model based on observations nested within subjects, subjects nested within units and units within forensic settings will be evaluated. Models will be compared according to relevant indices of fit, such as AIC, BIC and Log-Likelihood. To test for group differences, post hoc-independent *t*-test will be performed at all time-points. Effect sizes will be expressed in Cohen's d.

In answering the second primary research objective stated above, pertaining to the influence (or non-influence) of potential moderating factors on treatment outcomes, multiple linear regression will be used as an analysis tool.

In regards to the secondary research objective, exploring the experiences of staff administering IMR, a mixed methods approach will be employed. In analyzing quantitative data from the S-NoMAD and change and stability over time on the four indexes of this instrument, linear mixed models will be employed. In analyzing data from the semi-structured interviews with staff, a qualitative approach will be employed.

## 3 Ethics

In accordance with the relevant laws and regulations for studies with human subjects, the study has been approved by the Swedish Ethical Review Authority (Registration No. 2020–02046). One important aspect of the ethics pertaining to the study is the vulnerable nature of the individuals composing the study population. In addition to suffering from serious mental illness and being subject to strict regulation of everyday life, forensic mental health patients are also subject to various forms of stigma [38]. Thus, the study outlined in this article will take place in a context where power relations is an irrefutable and centrally influential feature of the milieu and the studied participants will be drawn from a population which is a highly marginalized group in society, often with limited ability to protect their own rights. From an ethical standpoint, these circumstances motivate an extra vigilant focus on ensuring that participating patients are informed of their rights as research participants and given ample opportunities to exercise them. In addition to participating patients, staff at the active wards will also be recruited to participate in the study for the purpose of answering the secondary objective pertaining to exploring experiences of working with the intervention. However, it seems highly reasonable to view staff participating in the study as less vulnerable than participating patients, given that the latter group will be comprised of persons with an anticipated low level of functioning that are also subject to highly restrictive state sanctioned limitations on autonomy and participation in society. Nevertheless, as in all studies, it will be important to inform staff approached for participation about their rights and the voluntary nature of research participation. In addition, we will ensure that staff are informed that the research team is independent from their employer.

The previously mentioned marginalized and stigmatized situation of the population of interest for this study, underscores the need to collect and store data in a judicious way. Data in a digital format will be encrypted and password protected. Physical copies such as completed questionnaires and consent forms will be stored in locked and secure conditions designed for storing research data securely (i.e. university facilities intended for this purpose). We will avoid collecting excessive and personal data, for instance by asking participants not to use their full name in any of the recordings of an IMR-session or during the S-NoMAD interviews.

In summary, ensuring and protecting the rights and interests of participating patients and staff is crucial. However, our estimation is that the measures described above minimize such risk. Given the almost non-existent evidence-base available in the forensic mental health field and the potential role of research in addressing important clinical issues, thus improving the care received by the individual patient, we argue that the benefits by far outweigh the potential risks.

## 4 Discussion

The proposed study will investigate the effects of an evidenced-based treatment within a population of offenders with serious mental illness. Thus, it relates to the issue of translatability of interventions used in non-forensic mental health care to forensic populations, which has been highlighted as a research field of practical and theoretical importance [1]. As previously mentioned, more evidence-based interventions within the field of forensic mental health care are sorely needed. This point is given further pertinence when the severe suffering of the patient population and potentially large societal ramifications of treatment failure are considered. In exploring experiences of staff delivering the intervention, as well as investigating the potential role of moderating factors on treatment outcome, the proposed study also aims to address questions of central importance for health-care providers making treatment decisions and organizing treatment delivery for complex patients with competing clinical needs. However, the proposed project has some important limitations that we feel deserve mentioning.

Firstly, there are differences between how countries structure forensic mental health care and how mental illness is considered in different judicial systems. Thus, when considering the generalizability of the results of our study to another national context, differences in national jurisprudence and practice of forensic mental health delivery should be taken into account. Secondly, the practitioners delivering IMR in our study will, as mentioned, be forensic mental health nurses and auxiliary staff at the wards in our active condition. They may have limited previous experiences with delivering structured psychosocial interventions. Thus, it is plausible that better quality of treatment could be insured by assigning treatment delivery to staff with more experiences of group treatments, e.g. a licensed psychologist well versed in IMR-treatment. However, previous studies with IMR have employed a similar method of delivery, with regular staff serving as group leaders after receiving training in IMR [11,39,40]. Our experience is that this is the usual mode of delivery for psychosocial interventions when applied within inpatient settings. Thus, we consider this aspect of our design to be a part that strengthens the ecological validity of the study. Furthermore, we have tried to compensate for this potential weakness by providing clinical supervision and by inclusion of process measures within our design.

Lastly, we would have wished to be able to include a larger number of participants within our study, to ensure sufficient statistical power. In planning our study, we have found it necessary to balance a desire for sufficient sample size against practical considerations based on finite resources and environmental constraints. Thus, results from outcome measures will have to be interpreted and reported with caution. However, our proposed sample size is comparable or markedly larger than samples included in several previous studies of IMR [[16], [39], [40]]. It should also be noted that the current project is not solely concerned with treatment outcomes but also pertain to staff experiences of implementing and working with IMR, which can hopefully add to our understanding of interventions of this kind when applied in this context. We also hope that our study can stimulate further enquiry into the effectiveness of psychosocial interventions within the forensic mental health field and ideally support meta-analytical efforts within the area.

## Funding statements

Region Dalarna, a government county in Sweden, funded parts of this work.

## CRediT authorship contribution statement

**Peter Andersson:** Conceptualization, Methodology, Writing – original draft, Writing – review & editing. **Malin Tistad:** Methodology, Supervision, Writing – review & editing. **Åsa Eriksson:** Supervision, Writing – review & editing. **Pia Enebrink:** Methodology, Supervision, Writing – review & editing. **Knut Sturidsson:** Conceptualization, Methodology, Supervision, Writing – review & editing.

## Declaration of competing interest

The authors have no competing interests to disclose.
